# The added value of (1,3)-β-D-glucan for the diagnosis of Invasive Candidiasis in ICU patients: a prospective cohort study

**DOI:** 10.1007/s15010-023-02053-4

**Published:** 2023-06-15

**Authors:** Martin Christner, Beya Abdennadher, Dominic Wichmann, Stefan Kluge, Amra Pepić, Martin Aepfelbacher, Holger Rohde, Flaminia Olearo

**Affiliations:** 1 Center for Diagnostics, Institute of Medical Microbiology, Virology and Hygiene, University Medical Center Hamburg-Eppendorf, Hamburg, Germany; 2 Center for Anesthesiology and Intensive Care Medicine, Department of Intensive Care Medicine, University Medical Center Hamburg-Eppendorf, Hamburg, Germany; 3 Center for Experimental Medicine, Institute of Medical Biometry and Epidemiology, University Medical Center Hamburg-Eppendorf, Hamburg, Germany

**Keywords:** (1,3)-β-D-glucan, Invasive Candidiasis, Intensive care unit, Diagnosis

## Abstract

**Purpose:**

Beta-D-Glucan (BDG) testing has been suggested to support the diagnosis of candidemia and invasive candidiasis. The actual benefit in critically ill high-risk patients in intensive care units (ICU) has not been verified so far.

**Methods:**

In ICU patients receiving empirical echinocandin treatment for suspected invasive candidiasis (IC), serial BDG testing using the Fujifilm Wako Beta-Glucan Test was performed, starting on the first day of echinocandin administration and every 24-48 h afterwards. Diagnostic accuracy was determined for single testing and serial testing strategies using a range of cut-off values. In addition, we compared the added value of these testing strategies when their results were introduced as additional predictors into a multivariable logistic regression model controlling for established risk factors of IC.

**Results:**

A total of 174 ICU patients, forty-six of which (25.7%) classified as cases of IC, were included in our study. Initial BDG testing showed moderate sensitivity (74%, 95%CI 59–86%) and poor specificity (45%, 95% CI 36–54%) for IC which could hardly be improved by follow-up testing. While raw BDG values or test results obtained with very high thresholds improved the predictive performance of our multivariable logistic regression model for IC, neither single nor serial testing with the manufacturer-proposed low-level cut-off showed substantial benefit.

**Conclusions:**

In our study of critically ill intensive care patients at high risk for candidemia or invasive candidiasis, diagnostic accuracy of BDG testing was insufficient to inform treatment decisions. Improved classification was only achieved for cases with very high BDG values.

**Supplementary Information:**

The online version contains supplementary material available at 10.1007/s15010-023-02053-4.

## Introduction

Invasive Candidiasis (IC) is increasingly recognized as a significant cause of morbiditiy and mortality in Intensive Care Unit (ICU) patients [1]. Prompt initiation of empirical antifungal treatment is deemed pivotal in order to impact patient survival [2]. However, the development of tools for the adequate diagnosis of IC has not kept pace with the burden of the disease and remains challenging. The gold standard histopathological detection of fungi in tissue specimens is often inapplicable [3] and the diagnostic value of routinely performed blood cultures [4] is reduced by low sensitivity and inherently slow turnaround times [5]. Consequently, a presumptive diagnosis of IC and initiation of empiric antifungal treatment is usually based upon a combination of clinical risk factors and microbiological criteria, e.g. the candida colonisation index (CI), resulting in low specificity and an excessive and/or unnecessary use of antifungals [6]. Biomarkers may support clinical decision-making by providing improved sensitivity and fast time-to-result, and in particular the (1 → 3)-β-D-glucan (BDG) assay has been advocated for this purpose in high-risk ICU patients [7]. However, reported sensitivities and specificities show substantial variation between studies [3, 7, 8], and it remains unclear if repeated BDG testing can improve diagnostic performance for IC.

The present study prospectively evaluated the diagnostic performance of the Fujifilm Wako BDG-test for the diagnosis of IC in high-risk ICU patients and analysed the added value of repeated testing and alternative cut-off values.

## Methods

### Patients and study design

This prospective observational cohort study was conducted from June 1 2020 to June 30 2021 in the Department of Intensive Care Medicine at the University Medical Center Hamburg-Eppendorf, Germany. The department consists of 12 ICUs with a total of 140 beds and serves all specialties of adult intensive care medicine.

In addition to routine diagnostic procedures, ICU physicians were instructed to order BDG testing upon initiation of empirical anti-fungal treatment for patients with high risk of IC and every 24-48 h thereafter for the total duration of antifungal therapy. ICU physicians were informed about the BDG test results, but were not specifically instructed to make any adjustments to the patient’s current therapy based on test results. All patients who received an echinocandin—the treatment of choice for suspected IC at our institution—and for whom at least one BDG measurement was available within 24 h of echinocandin administration were included in the study.

### Definitions

Cases of IC were identified based on criteria adopted from the revised and updated consensus definitions of invasive fungal disease from the European Organization for Research and Treatment of Cancer and the Mycoses study group education and research consortium (EORTC/MSGERC) [9]. To account for the higher risk of blood culture contamination in ICU populations [10] these criteria were modified as follows:

IC was defined as either (1) at least two positive blood cultures growing *Candida spp.*; or (2) detection of *Candida spp.* by direct microscopy or culture from a specimen obtained by a sterile procedure (including a drain placed within < 24 h) from a normally sterile site showing a clinical abnormality consistent with an infectious disease.

Patients with a single positive *Candida spp.* blood culture or with *Candida spp.* recovered from the wound, ascites, pleural puncture and/or intraabdominal drain site, along with clinical signs (leucocytosis > 12000 cells/mm^3^, hypothermia < 35.8 °C, fever > 38.5 °C and hypotension with MAP < 65 mmHg) were evaluated by two independent physicians, who used their clinical judgement to decide whether the patient should be classified as a case of IC.

### Microbiological analysis

Patient serum samples were tested for BDG using the Beta-Glucan Test (Fujifilm Wako Chemicals Europe, Neuss, Germany) following the manufacturer’s instructions. BDG levels were determined automatically using the MT-6500 toxinometer. Serum samples with BDG level over 600 pg/ml were diluted and retested. Results were evaluated using thresholds of 3, 7, 20, 50, 100, 150 and 200 pg/ml. If not stated otherwise, the manufacturer’s proposed cut-off of 7 pg/ml was used.

Blood culture bottles (Bactec Plus aerobic/anaerobic; BD, Heidelberg, Germany) were incubated in a BD Bactec Fx instrument (BD, Heidelberg, Germany) at 37° C for a maximum of five days. Fluid from positive blood culture bottles was microscopically evaluated and inoculated onto solid media, including a Sabouraud agar plate. Yeast from solid media subcultures was identified to the species level by MALDI-TOF mass spectrometry fingerprinting using a Bruker MALDI Biotyper system.

### Data collection

Demographic, clinical, and microbiological data were retrieved from clinical and laboratory information systems. Known risk factors for IC as well as basal clinical parameters were assessed, including age, sex, surgery, intestinal perforation, total parenteral nutrition, immunosuppression, transplantation, malignancy, presence of central venous catheters (CVC) or other vascular devices, dialysis, use of broad-spectrum antibiotics, length of ICU stay, leucocytes, C-reactive protein (CRP), procalcitonin (PCT), lactate, temperature, heart rate and respiratory rate. Overall severity of the disease was assessed by the SOFA score calculated on the first day of echinocandin treatment. The CI was determined as the ratio of the number of distinct body sites colonized by *Candida spp.* to the total number of cultured body sites [11] considering samples obtained between 10 days before echinocandin treatment and the end of the 90-day follow-up period. In addition, haemodialysis with cellulose membranes, immunoglobulin treatment [5, 12–14], albumin, antimicrobial treatment and concurrent bacteraemia [5] were assessed as potential risk factors for false positive BDG results.

Blood culture results were assessed immediately before, during and two weeks after treatment (except in case of death or discharge). Mortality was assessed after thirty days of follow-up.

The study protocol was approved by the Ethics Committee of the Hamburg Chamber of Physicians and a waiver of informed consent was granted (WF-19/20).

### Statistical analysis

Normally distributed continuous variables are presented as the mean ± standard deviation (SD), non‐normally continuous variables are presented as the median with interquartile range (IQR), and categorical variables are presented as numbers and proportions. Normal and non-normal distribution was established graphically. According to the distribution of the respective target outcome, differences between groups were assessed by t test, Mann–Whitney *U*-test, *χ*^ 2^ test or Fisher exact test as required. The diagnostic performance of BDG testing with different cut-off values (3, 7, 20, 50, 100, 150 and 200 pg/ml) and multiple-testing strategies (at least one test positive, all tests positive) was evaluated in terms of sensitivity and specificity as well as positive and negative predictive values for our study population. To evaluate the added value of BDG testing in the clinical pathway, logistic regression models based on clinically relevant judgment were calculated with IC state (yes vs. no) as a dependent variable and the following risk factors as predictors: age, sex, CI, parental nutrition, carbapenem treatment, dialysis, surgery, SOFA score, intestinal perforation and immunosuppression [15, 16]. The gained diagnostic information in relation to the base model was quantified on the basis of likelihood ratios, Nagelkerke pseudo-R^2^-values and the variation of predicted probabilities of IC[17]. Model performance figures from internal validation and measures of added value were corrected for overfitting using bootstrapping [18].

All analyses are performed in an explorative manner. No adjustment for multiple testing was performed.

Statistical analysis was performed using R version 4.4.1 [19]. Results were reported following the STROBE guidelines [20].

## Results

### Clinical characteristics

**Fig. 1:**
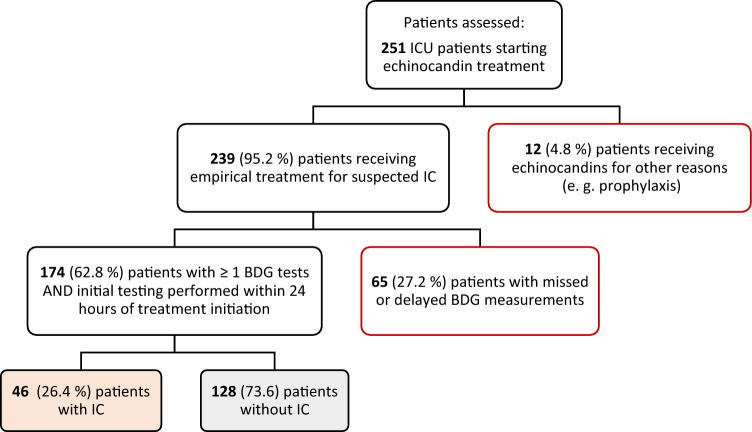
Study population and patient classification

During the study period 174 of 239 high-risk ICU patients receiving empirical echinocandin treatment for suspected IC were tested at least once for BDG (Fig. 1). The median age was 63 years (IQR 50–71) and participants were predominately male (130/174, 75.7%). Forty-six patients (26.4%) were diagnosed with IC, 35 (20.1%) with repeatedly positive blood cultures. Patients with and without IC had similar characteristics in baseline variables (Table 1). However, patients without IC were more likely to be SARS-CoV-2 infected (28.1% vs 4.3%, p-value = 0.002) and had a higher 30-day mortality (39.8% vs 21.7%, *p*-value = 0.023).

**Table 1:** Clinical characteristics of patients with and without invasive candidiasis (IC)

Characteristic	IC (*n* = 46)	No IC (*n* = 128)	*P* value
Age [years], median (IQR)	64 (53–73)	62 (49–70.5)	0.135
Sex, female (%)	11 (23.9)	33 (25.8)	0.958
Candida species in blood culture (no. of cases)	*C. albicans* (23)	*C. albicans* (2)^d^	–
	*C. parapsilosis* (4)	*C. glabrata* (1)^d^	
	*C. glabrata* (2)	*C. dubliniensis* (1)^d^	
	*C. dubliniensis* (2)		
	*C. tropicalis* (2)		
	*C. luisitaniae* (1)		
	*C. kefyr* (1)		
Candida Colonisation Index (IQR)	0.5 (0.3–0.7)	0.4 (0.2–0.7)	0.169
Underlying disease, no. of patients (%):			
Surgery^a^	27 (58.7)	58 (45.3)	0.128
Intestinal perforation	7 (15.2)	13 (10.2)	0.513
Solid tumor	6 (13.0)	31 (24.2)	0.168
Solid organ transplantation	2 (4.3)	16 (12.5)	0.092
Hematologic malignancy/HSCT	7 (15.2)	14 (10.9)	0.445
Dialysis	14 (30.4)	52 (40.6)	0.296
SARS-CoV-2 infection	2 (4.3)	36 (28.1)	**0.002**
SOFA score, media (IQR)	9.0 (7.0–12.0)	11.0 (8.0–13.25)	**0.014**
Parental nutrition (%)^1^	38 (84.4)	114 (89.1)	0.581
Central venous catheter (%)	45 (97.8)	124 (96.9)	0.733
Immunoglobulin treatment^a^ [-7/0 days] (%)	1 (2.2)	6 (4.7)	0.759
Bacteremia [-15/0 days] (%)	27 (58.7)	57 (44.5)	0.140
Antibiotic treatment^b^ (%):	37 (80.4)	110 (85.9)	0.518
Carbapenem treatment^b^ (%):	27 (58.7)	81 (63.3)	0.709
PCT [ng/l]^c^, median (IQR)	0.99 (0.44–2.02)	1.58 (0.56–4.2)	0.112
CRP [mg/liter], median (IQR)	119 (69.0–213.8)	154.5 (88–228)	0.263
Leucocytes [× 10^4^/ml], median (IQR)	12.1 (8.1–17.4)	14.2 (9.2–20.9)	0.337
Lactate [mmol/L], median (IQR)	1.3 (1.0–2.1)	1.6 (1.1–2.6)	0.139
Echinocandin treatment [days], median (IQR)	11.5 (5.3–16.8)	10.0 (5.0–15.0)	0.456
ICU stay [days], median (IQR)	30.5 (15.0–52.8)	27.0 (16.8–47.0)	0.748
30-day mortality [no. of deceased patients] (%)	10 (21.7)	51 (39.8)	**0.043**

^a^One missing value in the group of patients with IC

^b^During the 1st week of echinocandin treatment

^c^5 missing values in the group of patients with IC, 9 missing values in the group of patients without IC

^d^Singular positive blood cultures from patients with at least 2 additional negative blood cultures obtained within 72 h before the allegedly positive sample and candida colonization index ≥ 0.5; considered a contaminant

### Results from BDG testing

Upon initial testing, patients with IC showed higher levels of serum BDG and higher test positivity rates than patients without IC. These differences persisted in follow-up tests (table S1). Discrepant results upon follow-up testing were obtained from 16 patients (4 with IC) with initially positive and 22 patients (0 with IC) with initially negative BDG test results. The variation of BDG values in these patients followed different patterns (single outlier, low amplitude fluctuation, high amplitude fluctuation, gradual increase/decrease, discernible peak), not suggestive of a single source of bias.

### Diagnostic accuracy of BDG testing

Applying the manufacturer’s cut-off, BDG testing showed moderate sensitivity (74%, 95%CI 59–86%) and poor specificity (45%, 95% CI 36–54%) for IC in our population of high-risk ICU patients. Diagnostic accuracy could hardly be improved by repeated testing alone and specificity and positive predictive value remained poor even when considering multiple consecutive positive tests. Satisfactory sensitivity or specificity could only be obtained with adjusted cut-offs (Table 2). However, the introduction of an indeterminate category to minimize the number of false positive and false negative calls in our patient population (e. g. < 3 pg/ml <  = indeterminate < 100 pg/ml), would render the assay meaningless in more than 70% of our samples (table S2).

**Table 2:** Diagnostic performance of (1,3)-β-d-glucan testing for IC

Algorithm	Cut-off [pg/ml]	Sensitivity (95% CI) [%]	Specificity (95% CI) [%]	PPV (95% CI) [%]	NPV (95% CI) [%]
Single test (*n* = 174)	3	87 (74–95)	27 (19–35)	30 (22–38)	85 (70–94)
	7^1^	74 (59–86)	45 (36–54)	33 (24–43)	83 (72–91)
	20	48 (33–63)	69 (60–77)	35 (24–49)	79 (70–86)
	50	30 (18–46)	89 (82–94)	50 (31–69)	78 (70–84)
	100	17 (8–31)	95 (90–98)	57 (29–82)	76 (69–83)
	200	17 (8–31)	100 (97–100)	100 (63–100)	77 (70–83)
2 tests, ≥ 1 pos. (n = 148)	3	92 (79–98)	19 (12–28)	29 (21–38)	88 (68–97)
	7^1^	77 (61–89)	32 (23–42)	29 (20–39)	80 (65–90)
	20	54 (37–70)	57 (47–66)	31 (20–43)	78 (67–86)
	50	36 (21–53)	81 (72–88)	40 (24–58)	78 (69–85)
	100	21 (9–36)	90 (83–95)	42 (20–67)	76 (68–83)
	200	18 (8–34)	98 (94–100)	78 (40–97)	77 (69–84)
2 tests, 2 pos. (*n* = 148)	3	90 (76–97)	27 (19–36)	30 (22–40)	88 (72–97)
	7^1^	72 (55–85)	50 (40–59)	34 (24–45)	83 (72–91)
	20	46 (30–63)	74 (65–82)	39 (25–55)	79 (70–87)
	50	31 (17–48)	94 (88–98)	67 (41–87)	79 (71–86)
	100	18 (8–34)	97 (92–99)	70 (35–93)	77 (69–84)
	200	15 (6–31)	100 (97–100)	100 (54–100)	77 (69–83)
3 tests, ≥ 1 pos. (*n* = 120)	3	94 (80–99)	10 (5–19)	28 (20–38)	82 (48–98)
	7^1^	79 (61–91)	26 (18–37)	29 (20–39)	77 (58–90)
	20	61 (42–77)	47 (36–58)	30 (20–43)	76 (62–87)
	50	39 (23–58)	76 (65–84)	38 (22–56)	77 (66–85)
	100	24 (11–42)	91 (83–96)	50 (25–75)	76 (67–84)
	200	18 (7–35)	97 (90–99)	67 (30–93)	76 (67–83)
3 tests, 3 pos. (*n* = 120)	3	91 (76–98)	25 (17–36)	32 (22–42)	88 (69–97)
	7^1^	70 (51–84)	51 (40–61)	35 (24–48)	81 (69–91)
	20	42 (25–61)	74 (63–82)	38 (22–55)	77 (67–86)
	50	30 (16–49)	97 (90–99)	77 (46–95)	79 (70–86)
	100	18 (7–35)	99 (94–100)	86 (42–100)	76 (67–84)
	200	15 (5–32)	100 (96–100)	100 (48–100)	76 (67–83)

*PPV* Positive predictive value, *NPV* Negative predictive value, *n* Number of patients

^1^Manufacturer proposed cut-off

### The added value of BDG measurement

**Fig. 2:**
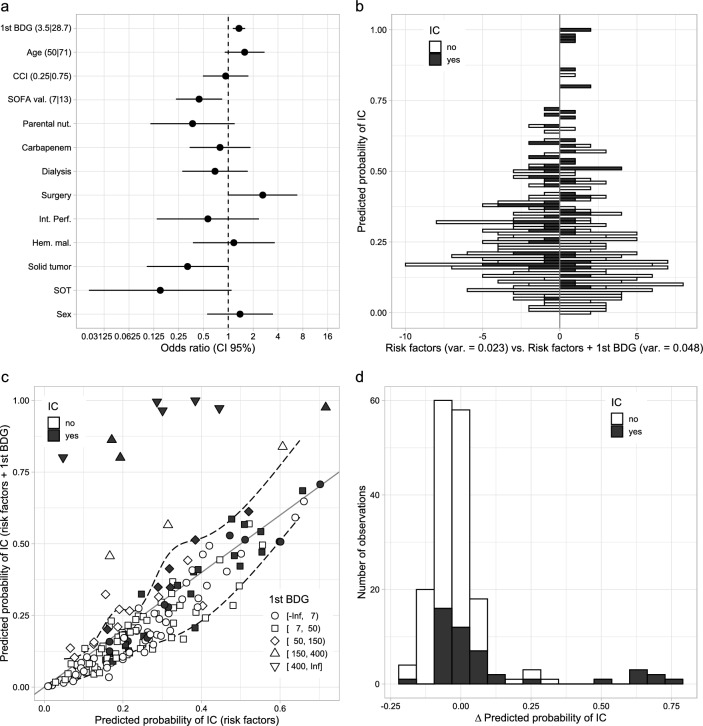
Panel **a**: Multivariable logistic regression model for invasive candidiasis (IC) using known risk factors and the BDG value from the first BDG test as predictors (model M3 from table S3). Dots and whiskers indicate odds ratios with 95% confidence intervals for changing the value of the respective predictor as indicated in square brackets. Panel **b**: Comparison of predicted probabilities for IC between the aforementioned model (‘1stBDG’, right) and the respective base model without BDG (‘Risk factors + BDG’, model M1 from table S3, left). Black fill indicates cases of IC. Panel **c**: Comparison of predicted probability for IC from models with (‘Risk factors + BDG’, y-axis) and without (‘Risk factors’, x-axis) BDG. Black fill indicates cases of IC; symbols indicate BDG levels. Panel **d**: Distribution of differences in the predicted probability of IC between the aforementioned models. Black fill indicates cases of IC. Substantial changes in the predicted probability of IC are limited to cases with very high BDG values (as compared to the manufacturer’s proposed cut-off of 7 pg/ml)

**Fig. 3:**
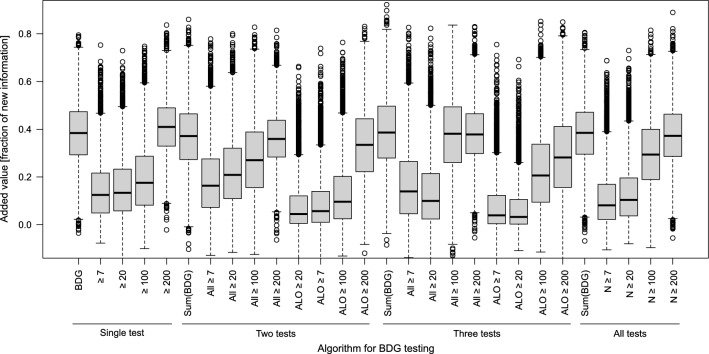
Added value of BDG testing using different cut-off values and multiple-testing strategies (single test: BDG value or positivity using the indicated cut-offs, two/three tests: sum of BDG values, positivity of all or at least one (ALO) test using the indicated cut-offs; All tests: sum of BDG values or number (*N*) of positive tests using the indicated cut-offs). Boxplots show the fraction of information gained from introducing results from BDG testing as an additional predictor to a pre-specified model with established risk factors for IC (age, sex, CI, parental nutrition, carbapenem treatment, dialysis, surgery, SOFA score, intestinal perforation and hematologic malignancy) with median (horizontal lines), IQR (box) and 1.5 × IQR (whiskers) from bootstrapping with 10.000 replicates. The fraction of gained diagnostic information was quantified on the basis of changes in the variation of predicted probabilities of IC [Frank E Harrell, Jr. Regression Modeling Strategies. 2nd ed. 2015. Springer Cham. https://doi.org/10.1007/978-3-319-19425-7]

The prognostic value of BDG testing was also assessed by analyzing changes in the performance of logistic regression models after the addition of BDG test results as an additional predictor of IC. The addition of raw BDG values from initial testing to a pre-specified multivariable regression model, encompassing previously described risk factors for IC as predictors of IC, improved model fit and performance (likelihood ratio *χ*2 = 22.1, d. f. = 1, *P* < 0.001; table S3) and widened the histogram of predicted probability as would be expected for a predictor which adds clinically important predictive information (Fig. 2b). However, in line with the observed distribution of IC cases over BDG values (table S2), substantial changes in the predicted probability of IC were limited to a small number of samples with very high BDG values (Fig. 2c). Thus BDG testing increased the likelihood of correct classification only for a small fraction of cases. Not surprisingly, as the increase in predictive performance was attributable to samples with the highest BDG values, the application of low-level thresholds, as usually employed for BDG testing, markedly reduced the fraction of new information gained from BDG test results (Fig. 3). As already observed for diagnostic accuracy, the incorporation of results from follow-up testing only marginally affected BDG’s added value. None of the investigated multi-test regimen added more information than raw BDG values from initial testing (Fig. 3).

## Discussion

With the high mortality of IC in ICU patients fast and reliable diagnostic tests are highly appreciated. BDG holds the promise to improve the diagnostic yield, turnaround and accuracy in IC. However, this hope was not fulfilled in our cohort of 174 critically ill patients with high risk for IC. Although our data support the suitability of BDG as a predictor of IC, the observed diagnostic accuracy using recommended thresholds was low and could hardly be improved by repeated testing. Thus adding BDG testing to the diagnostic algorithm, even if done as serial testing, which has been suggested to improve the diagnostic accuracy compared to single testing [7], cannot be recommended for ICU patients with a high risk of IC. Our study found low specificity and low positive predictive value for BDG testing with recommended cut-offs in the respective patient population. These findings were supported by logistic regression modelling, which found limited added value of single or repeated BDG testing with low-level thresholds. On the other hand, the sensitivity and negative predictive value, even of serially negative tests, seem to be insufficient to mandate discontinuation of empirical antifungal treatment in high-risk individuals until the implications of numerous plausible reports on BDG negative IC cases are better understood [21][21].

A common problem with biomarkers in fungal diagnostics is the setting of appropriate cut-offs. While such categorization ensures easily comprehensible reports, it is associated with the loss of information and might cause severe heterogeneity across patient populations[3]. In our study, the application of the recommended thresholds—which might be appropriate for non-ICU patients [7]—resulted in a severe loss of predictive performance as compared to raw BDG values. However, strategies to counteract the apparently reduced discriminatory power of BDG in our patient population with separate high- and low-level thresholds to rule in or out IC would render BDG testing uninformative for the majority of patients. While careful selection of patients for BDG testing has been shown to improve the test’s predictive performance, restrictions on test utilization are difficult to enforce in clinical practice.

Our insights come with some limitations. The study is monocentric and focused on high-risk ICU patients. Findings do thus not extend to non-ICU populations and individuals with low to moderate risk of IC. As our definitions for IC in high-risk ICU patients were not identical to the EORTC/MSGERC revised and updated consensus definitions of IFD from 2020 our results might not be directly comparable to studies applying these widely used definitions. Generalizability might be further hampered by differences in diagnostic performance between the various BDG tests available. However, direct comparisons involving the Fujifilm Wako BDG assay and the Associates of Cape Cod Fungitell assay (which is part of the EORTC/MSGERC) showed good agreement between both tests [23, 24] The small sample size limits the power of our statistical analyses. The number of available cases for certain analyses was further reduced by the fact that not all patients received multiple BDG tests. Although these limitations resulted in wide confidence intervals for some estimates, the observed trends were robust and consistent across different analyses. Furthermore, we are aware of the possible gradual loss of sensitivity of BDG testing during ongoing echinocandin treatment. However, such loss of sensitivity has not yet been addressed in clinical studies and might be more relevant after prolonged treatment. Among our patients with discrepant results upon follow-up testing, increasing and decreasing BDG levels were observed in equal proportions. Considering the high mortality of IC in ICU patients and the high pre-test probability in our patients with established risk factors, it would have been unethical to delay the start of treatment awaiting results from serial BDG testing. Finally, our study was conducted during the COVID-19 pandemic which probably affected various aspects of our venture. Staff overload impaired compliance with our study protocol resulting in an unexpectedly high number of exclusions due to delayed or missing initial BDG measurements and undesirable trade-offs regarding the collection of samples for the determination of the CCI. In addition, the observed negative correlation between SARS-CoV-2 infection and IC might have obfuscated other typical IC associations (e. g. with SOFA score or mortality).

In conclusion, due to low specificity and moderate sensitivity, BDG testing with recommended cut-offs has limited value to guide treatment decisions in high-risk ICU patients even with costly and time-consuming repeated measurements. The clinical impact of improved performance observed with adapted thresholds or quantitative assessment might warrant further investigation. Future outcome-orientated studies should thus comparatively address the added value of available IC markers to guide the initiation or discontinuation of antifungal treatment in defined patient populations.

### Supplementary Information

Below is the link to the electronic supplementary material.

Supplementary file1 (PDF 534 KB)

## Data Availability

Not applicable.
