# Supplementary material for: The added value of (1,3)-β-D-glucan for the diagnosis of Invasive Candidiasis in ICU patients: a prospective cohort study
Source: Infection. 2023 Jun 15;52(1):73–81. doi: 10.1007/s15010-023-02053-4 (PMC10811116; doi:10.1007/s15010-023-02053-4)
Supplement: Supplementary file 1 — Supplementary file1 (PDF 534 KB) [file 15010_2023_2053_MOESM1_ESM.pdf]

The added value of (1,3)-β-D-glucan for the diagnosis of Invasive Candidiasis in ICU patients: a prospective cohort study - Supplemental information

Martin Christner[1], Beya Abdennadher[1], Dominic Wichmann[2], Stefan Kluge[2], Amra Hot[3], Martin Aepfelbacher[1], Holger Rohde[1], Flaminia Olearo[1]

1. Center for Diagnostics, Institute of Medical Microbiology, Virology and Hygiene, University Medical Center Hamburg-Eppendorf, Hamburg, Germany.

2. Center for Anesthesiology and Intensive Care Medicine, Department of Intensive Care Medicine, University Medical Center Hamburg-Eppendorf, Hamburg, Germany.

3. Center for Experimental Medicine, Institute of Medical Biometry and Epidemiology, University Medical Center Hamburg-Eppendorf, Hamburg, Germany.

Table S1: Characteristics of the first three BDG tests per patient

Test	Characteristic	With proven IC	Without proven IC
1st BDG test (n = 174; 46 with proven IC, 128 without proven IC)	Median BDG value [pg/ml] (IQR)	18.5 (5.6-58.3)	8.6 (2.9-24.5)
	Num. with BDG ≥ 3 (Prop. [%])	40 (87.0)	94 (73.4)
	Num. with BDG ≥ 7 (Prop. [%])	34 (73.9)	70 (54.7)
	Num. with BDG ≥ 20 (Prop. [%])	22 (47.8)	40 (31.3)
	Num. with BDG ≥ 50 (Prop. [%])	14 (30.4)	14 (10.9)
	Num. with BDG ≥ 100 (Prop. [%])	8 (17.4)	6 (4.7)
	Num. with BDG ≥ 200 (Prop. [%])	8 (17.4)	0 (0.0)
2nd BDG test (n = 148; 39 with proven IC, 109 without proven IC)	Median BDG value [pg/ml] (IQR)	18.7 (5.8-78.1)	10.6 (3.6-26.7)
	Num. with BDG ≥ 3 (Prop. [%])	36 (92.3)	86 (78.9)
	Num. with BDG ≥ 7 (Prop. [%])	28 (71.8)	69 (63.3)
	Num. with BDG ≥ 20 (Prop. [%])	19 (48.7)	38 (34.9)
	Num. with BDG ≥ 50 (Prop. [%])	14 (35.9)	15 (13.8)
	Num. with BDG ≥ 100 (Prop. [%])	14 (35.9)	15 (13.8)
	Num. with BDG ≥ 200 (Prop. [%])	6 (15.4)	0 (0.0)
3rd BDG test (n = 120; 33 with proven IC, 87 without proven IC)	Median BDG value [pg/ml] (IQR)	29.7 (6.5-80.0)	11.8 (5.1-33.3)
	Num. with BDG ≥ 3 (Prop. [%])	31 (93.9)	73 (83.9)
	Num. with BDG ≥ 7 (Prop. [%])	55 (72.7)	24 (63.2)
	Num. with BDG ≥ 20 (Prop. [%])	18 (54.6)	38 (43.7)
	Num. with BDG ≥ 50 (Prop. [%])	12 (54.5)	12 (13.8)
	Num. with BDG ≥ 100 (Prop. [%])	7 (21.2)	5 (5.7)
	Num. with BDG ≥ 200 (Prop. [%])	5 (15.2)	3 (3.5)

Table S2: Distribution of initial BDG values in patients with and without IC

BDG value (1st test)	Number (proportion [%]) of patients with IC	Number (proportion [%]) of patients without IC
[0, 3)	6 (13.0)	34 (26.6)
[3, 7)	6 (13.0)	24 (18.8)
[7, 20)	12 (26.1)	30 (23.4)
[20, 50)	8 (17.4)	26 (20.3)
[50, 100)	6 (13.0)	8 (6.2)
[100, 200)	0 (0.0)	6 (4.7)
[200, 1000)	8 (17.4)	0 (0.0)
Total	46	128

Table S3: Multivariable logistic regression models for IC.

	M1: Risk factors		M2: 1st BDG ≥ 7 pg/ml		M3: 1st BDG value	
**Predictor**	**β**	**SE**	**β**	**SE**	**β**	**SE**
1st BDG ≥ 7 pg/ml (1 = yes, 0 = no)	-	-	1.024**	0.450	-	-
1st BDG value [pg/ml]	-	-	-	-	0.012***	0.003
CCI	0.361	0.591	0.253	0.604	-0.147	0.646
Parental nutrition (1 = yes, 0 = no)	- 0.711	0.577	- 0.801	0.591	-0.997*	0.599
Carbapenem treat. (1 = yes, 0 = no)	- 0.100	0.395	- 0.094	0.402	-0.231	0.432
SOFA score	- 0.113**	0.049	- 0.142 ***	0.052	-0.136**	0.055
Dialysis (1 = yes, 0 = no)	- 0.116	0.417	- 0.228	0.429	-0.371	0.466
Surgery (1 = yes, 0 = no)	0.684	0.457	0.593	0.466	0.961*	0.492
Intestinal perf. (1 = yes, 0 = no)	0.077	0.603	- 0.038	0.619	-0.569	0.727
Hem. mal. (1 = yes, 0 = no)	0.052	0.565	0.259	0.573	0.154	0.582
Solid tumor (1 = yes, 0 = no)	- 1.231**	0.543	- 1.031 *	0.549	-1.136*	0.580
Solid organ trans. (1 = yes, 0 = no)	- 1.270	0.835	- 1.102	0.853	-1.898*	1.015
Age [year]	0.022*	0.013	0.021	0.013	0.022	0.013
Sex (1 = male, 0 = female)	0.238	0.432	0.258	0.446	0.327	0.468
Intercept	- 0.910		- 1.164	0.990	-0.674	1.002
Number of observations	174		174		174	
Model likelihood ratio (χ^2, d. f., P)	21.5, 12, 0.041		27.0, 13, 0.012		43.6, 13, <0.001	
Goodness of fit test[1] (Z, P)	-0.136, 0.892		-0.304, 0.761		-0.167, 0.867	
Nagelkerke R^2 (bias corrected[2])	0.169 (0.012)		0.210 (0.043)		0.323 (0.163)	
C-statistic (bias corrected[2])	0.710 (0.616)		0.737 (0.645)		0.782 (0.709)	
Akaike's information criterion	205.5		202.0		185.4	

1) S. le Cessie & J. C. van Houwelingen. A Goodness-of-Fit Test for Binary Regression Models, Based on Smoothing Methods. Biometrics Vol. 47, No. 4 (Dec., 1991). DOI: https://doi.org/10.2307/2532385

2) Frank E Harrell, Jr. Regression Modeling Strategies. 2nd ed. 2015. Springer Cham. DOI: https://doi.org/10.1007/978-3-319-19425-7

CCI: candida colonization index, Hem. mal.: hematologic malignancy, trans.: transplantation, treat.: treatment
